# Validity and Reliability of the Japanese Version of the Frontal Assessment Battery in Patients with Stroke

**DOI:** 10.3390/neurolint16050081

**Published:** 2024-09-25

**Authors:** Katsuya Sakai, Yuichiro Hosoi, Yusuke Harada, Kenji Morikawa, Yuichi Kato

**Affiliations:** 1 Department of Physical Therapy, Faculty of Health Sciences, Tokyo Metropolitan University, Tokyo 116-8551, Japan; 2 Department of Rehabilitation Medicine, Keio University School of Medicine, Tokyo 160-8582, Japan; s1455105@sc.sozo.ac.jp; 3 Department of Sports Health Sciences, Ritsumeikan University, Kyoto 525-8577, Japan; 4 Department of Rehabilitation, Reiwa Rehabilitation Hospital, Chiba 260-0026, Japan; harada1111.0917@gmail.com; 5 Department of Rehabilitation, Ishikawajima Memorial Hospital, Tokyo 104-0051, Japan; ptkmorikawa@gmail.com; 6 Graduate School of Human Health Sciences, Tokyo Metropolitan University, Tokyo 116-8551, Japan; yu1ii10122101@outlook.com; 7 Department of Rehabilitation, Moriyama Neurological Center Hospital, Tokyo 134-0088, Japan

**Keywords:** frontal assessment battery, Japanese version, stroke patients, reliability, validity

## Abstract

Background: The Frontal Assessment Battery (FAB), which is used to assess executive function, has been translated into several languages and shown to be valid and reliable. However, the validity and reliability of the Japanese version in patients with stroke are unknown. This study aimed to investigate the validity and reliability of the Japanese version of the FAB in patients with stroke. Methods: The Japanese version of the FAB for dementia was modified and evaluated in 52 patients with stroke. FAB measurements were obtained twice over a 10-day period. Convergent validity was assessed using the Stroop Color Word Test (SCWT) and the Trail Making Test (TMT) part B. Internal consistency was measured using Cronbach’s alpha (Cα). Test-retest evaluations were performed using intraclass correlation coefficient [ICC (2.1)] measurements, and limits of agreement (LOA) were calculated using the total FAB score. Results: The mean total FAB score was 13.4 ± 2.8 points, the ICC (2.1) was 0.856, and Cα was 0.92. The total FAB score was correlated with SCWT scores for parts I through IV (r = 0.70 to 0.77) and the TMT score for part B (ρ = −0.53). The LOA were −1.7 to 2.9 points. Conclusions: The Japanese version of the FAB had higher validity and reliability in patients with stroke.

## 1. Introduction

Higher brain dysfunction has a significant impact on the activities of daily living (ADLs) and return to society in patients with mild cognitive impairment and stroke [1,2,3]. In particular, executive dysfunction occurs in approximately 25–75% of patients with stroke [3,4]. Executive dysfunction is characterized by difficulty in planning actions, making decisions, thinking flexibly, and initiating actions, and it impairs the ability to perform basic and instrumental ADLs, such as shopping, cooking, and calling [3,5]. Lipskaya-Velikovsky et al. [5] investigated the relationship between executive dysfunction and basic and instrumental ADL function in patients with stroke. They reported that executive dysfunction was correlated with basic and instrumental ADL function in patients with stroke. In addition, Sakai et al. [6] conducted a cluster analysis using the Trail Making Test (TMT) part B as an executive function test to classify executive dysfunction by severity and to analyze differences in walking and basic ADL function. The TMT part B is a test of executive function that involves connecting numbers and letters in alternating order [7]. The frontal lobe is the brain region involved in performing the TMT part B [7]. In their study, executive dysfunction was classified into three clusters: mild, moderate, and severe. Executive dysfunction was associated with walking and basic ADL function in patients with stroke. Sakai et al. [6] found a cluster of patients with high walking ability but impaired executive function, in which basic ADL function was clearly impaired. Thus, executive dysfunction is associated with walking and basic ADL function in patients with stroke, which limits their reintegration into society.

The existing tools for assessment of executive dysfunction include the TMT [7], Frontal Assessment Battery (FAB) [8], behavioral assessment of dysexecutive syndrome [9], and the Stroop Color Word Test (SCWT) [10]. The TMT, SCWT, and FAB are commonly used to assess rehabilitation of executive dysfunction in patients with stroke [11]. This is because the TMT, SCWT, and FAB have short measurement times, are less burdensome for the patients, and are more likely to capture executive dysfunction in patients with stroke [8,9,10,11].

The FAB is an assessment tool created by Dubois et al. to evaluate frontal lobe and executive function [8]. The advantage of the FAB is that it divides executive functions into six categories (conceptualization, mental flexibility, motor programming, sensitivity to interference, inhibitory control, and environmental autonomy), each of which can be assessed individually. The original version of the FAB was associated with other executive and cognitive function assessments and was highly valid and reliable [8]. The Japanese version of the FAB was created by Kugo et al. [12] for patients with dementia and exhibits high validity and reliability. However, the validity and reliability of the Japanese version of the FAB have not been tested in patients with stroke, despite its frequent use as a rehabilitation assessment of executive function in patients with stroke.

In addition, at present, no indicator is available to determine whether interventions such as rehabilitation improve executive function beyond the range of measurement errors. Several methods have been recently developed for calculating values above the measurement error or acceptable range of error, such as the minimal detectable change (MDC), minimal important change (MIC), and limits of agreement (LOA) [13,14]. By using MDC and MIC, it is possible to determine whether the difference between pre- and post-intervention binary measures is due to errors caused by multiple measurements or changes caused by the intervention, which may help in determining the effect of the intervention and in making decisions on treatment selection. However, the MDC, MIC, and LOA of the FAB have not been calculated. An index to determine the effectiveness of interventions such as rehabilitation for executive dysfunction and to determine whether the findings reflect an intervention effect or a measurement error is required. Therefore, the purpose of this study was to verify the validity and reliability of the Japanese version of the FAB in patients with stroke.

## 2. Materials and Methods

### 2.1. Participants

The study population included 52 patients with stroke (mean age: 72.6 ± 10.2 years; males, 32; females, 20; time since stroke: mean, 71.4 ± 63.8 days). This study was conducted at three rehabilitation hospital units between August 2022 and October 2023. The sample size was calculated with a power 0.80, α = 0.05, and correlation value = 0.654 [12] using G power 3.1.9.2, which indicated that the ideal sample size should be larger than 13 participants. The inclusion criteria were as follows: (1) first-time stroke, (2) age > 18 years, (3) absence of upper limb orthopedic disease, and (4) presence of hemiplegia. The exclusion criteria were as follows: (1) diagnosis of severe dementia and Alzheimer’s disease, (2) diagnosis of higher brain dysfunction (e.g., unilateral spatial neglect, aphasia, and apraxia), and (3) age > 90 years. Patients received 40 to 60 min of basic rehabilitation every day (e.g., standing exercises, balance exercises, walking exercises).

### 2.2. Adaptation of the Japanese Version of the Frontal Assessment Battery for Patients with Stroke

First, we contacted the author of the Japanese version of the FAB and obtained permission for its modification and adaptation for patients with stroke. The FAB consists of six items, each of which is scored from 0 to 3, with a total score of 18 points. Higher scores indicate higher executive and frontal lobe function [8]. The Japanese version of the FAB has been reported to be a valid and reliable test for patients with dementia [12]. The intraclass correlation coefficient (ICC (2.1)) of the Japanese version of the FAB was 0.972 (95% confidence interval (CI): 0.956–0.983), while its concurrent validity was −0.484–0.725 [12], indicating the reliability and validity of the Japanese version of the FAB. The FAB includes evaluation of motor performance in items 3 (Luria motor sequences), 4 (conflicting instructions), 5 (go-no-go test), and 6 (prehension behavior). Therefore, we modified the FAB for use on the non-paralyzed side (Supplementary Material S1).

### 2.3. Assessments

The FAB was administered twice (within 10 days), and other assessments were performed simultaneously.

Other executive and cognitive functions were assessed using the TMT parts A and B, the SCWT parts I–IV, and the Mini-Mental State Examination (MMSE). These assessments were conducted in a quiet room with the participants sitting in chairs. Other physical functions were assessed using the Brunnstrom recovery stage (BRS), stroke impairment assessment set (SIAS), and functional independence measure (FIM).

The TMT part A reflects motor speed and attention, whereas the TMT part B has often been used to assess executive function [7,15]. The TMT part B minus A reflects executive function [7,15]. Therefore, the TMT part B and B minus A were used in this study. For the TMT part A, the participants connected the circled numbers (1–25) in sequence as fast as possible [7,15]. In the TMT part B, participants connected numbers and letters alternately in sequence as quickly as possible [7,15].

The SCWT assesses executive function in four parts [10]. SWCT parts I to IV are each 40 s tests consisting of 30 pieces. Part I involves matching the names of colors written in black ink with the corresponding color. Part II involves matching the names of colors written in various colors with the corresponding color. Part III involves matching the names of colors written in various colors and black ink, and part IV involves matching words written in various colors with the names of the corresponding colors written in black ink. The SWCT calculates the correct and incorrect answers, and in this study, based on a previous study [12], the number of correct answers was used as a measure of executive function.

The MMSE was used to assess cognitive function, with total scores of 30 points indicating better cognitive function [16]. MMSE scores of 0–10, 11–20, 21–23 or higher, and ≥24 indicated severe cognitive impairment, moderate cognitive impairment, mild cognitive impairment, and normal cognition, respectively [16].

The BRS assesses the degree of motor paralysis of the upper limb, finger, and lower limb functions [17] and consists of six items.

The SIAS is a comprehensive tool for assessing motor and sensory functions in patients with stroke [18]. The SIAS, which consists of 22 items, classifies functional disorders into 9 types. This study used only the item corresponding to the grip strength on the non-paralyzed side. This test was rated on a 4-point scale (score range: 0–3). The better the non-paralyzed function, the higher the number of points.

The FIM is an assessment tool that measures the ability to perform ADLs [19] and consists of FIM motor items (13 items) and FIM cognitive items (5 items) [18]. Each item was rated using a 6-point scale (score range, 1–7), with 91, 35, and 126 being the highest scores for FIM motor, FIM cognitive, and FIM total, respectively. Higher scores indicate better functioning in ADLs.

### 2.4. Statistical Analysis

The Shapiro–Wilk test was used to determine the data distribution. The convergent validity between the FAB and the TMT part B, the TMT part B minus A, the SCWT parts I–IV, and MMSE scores was calculated using correlation coefficients. Correlation was categorized as negligible (0–0.10), weak (0.10–0.39), moderate (0.40–0.69), strong (0.70–0.89), and very strong (0.90–1.00) based on the corresponding correlation coefficient values [20]. Internal consistency was evaluated using Cronbach’s alpha (Cα). Cα values between 0.70 and 0.95 indicated strong internal consistency [21]. Test-retest reliability was evaluated using the ICC (2.1) and classified as follows based on ICC values: <0.5 = poor reliability; 0.5 to <0.75 = moderate reliability; 0.75–0.9 = good reliability; and >0.9 = excellent reliability [22]. The agreement between the first and second FAB total scores was checked for fixed and proportional errors using Bland–Altman analysis [23]. If fixed and proportional errors were not identified, MDC was calculated; if they were identified, the LOA were calculated [13]. The LOA were calculated using the formula LOA = mean the difference ± 1.96 × standard deviation of difference [13]. Statistical analysis was performed using SPSS (version 29.0; SPSS Inc., Chicago, IL, USA), and statistical significance was set at *p* < 0.05.

## 3. Results

A total of 52 patients with stroke participated in the study (mean age: 72.6 ± 10.2 years; males, 32; females, 20; mean time since stroke: 71.4 ± 63.8 days; Table 1). The SIAS, BRS, and FIM scores are presented in Table 1. The mean total FAB score was 13.4 ± 2.8 points, and the scores for FAB items 1–6 and other assessments are shown in Table 2.

The results of the Shapiro–Wilk test, total FAB score, and SCWT parts I and II were normally distributed (*p* > 0.05). Other variables were not normally distributed (*p* < 0.05).

In assessments of convergent validity (correlation coefficient), the total FAB score was significantly positively correlated with the SCWT scores for parts I–IV (part I: r = 0.738, *p* < 0.001, part II: r = 0.767, *p* < 0.001, part III: ρ = 0.724, *p* < 0.001, part IV: ρ = 0.673, *p* < 0.001, Table 3) and the MMSE score (ρ = 0.458, *p* = 0.001, Table 3). Furthermore, the total FAB score was significantly negatively correlated with the scores for the TMT part A (ρ = −0.389, *p* = 0.005, Table 3), the TMT part B (ρ = −0.622, *p* < 0.001, Table 3), and B minus A (ρ = −0.614, *p* < 0.001, Table 3). In assessments of reliability, ICC (2.1) was 0.856 (95% CI: 0.761–0.915), and the Cα value was 0.92.

**Figure 1:**
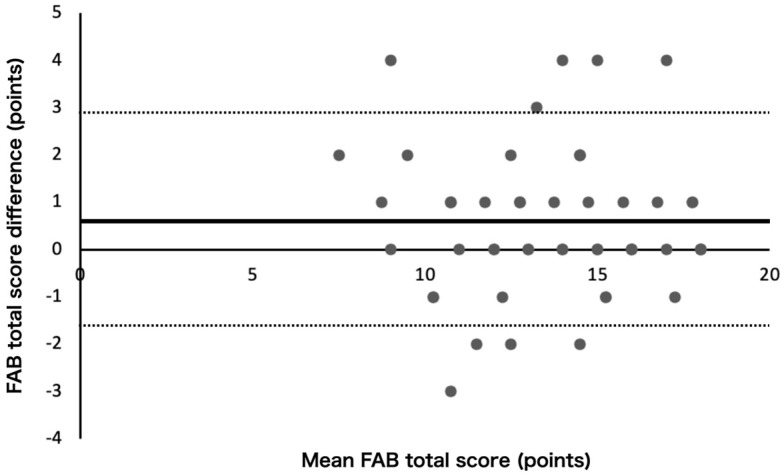
Bland–Altman plot of the Frontal Assessment Battery.

The Bland–Altman analysis showed that proportional errors were not observed (*p* > 0.05), whereas fixed errors were observed (t = −2.7, *p* < 0.05, 95% CI: −1.01 to −0.15). Therefore, the LOA were −1.7–2.9 points (Figure 1).

The thick solid line represents the mean difference between sessions, and the dashed lines represent the limits of agreement. The Bland–Altman plot shows fixed errors.

## 4. Discussion

This study modified the Japanese version of the FAB for patients with stroke, investigated its validity and reliability, and calculated the LOA. The Japanese version of the FAB showed strong internal consistency and good reliability for patients with stroke, with LOA of −1.7 to 2.9 points. This study indicates that the Japanese version of the FAB is a useful assessment tool for patients with stroke.

In assessments of convergent validity, the total FAB score of the Japanese version of the FAB for patients with stroke was moderately to strongly correlated with the SCWT scores for parts I to IV, the TMT parts B and B minus A scores, and the MMSE score. These results are consistent with those of previous studies [24,25]. Han et al. [24] conducted neuropsychological assessments using the FAB, TMT, and SCWT, and investigated whether these assessments were relevant in patients with stroke. They reported that the total FAB score correlated with the score for the TMT part B (r = −0.382) and the scores for the SCWT parts I–IV (part I: r = −0.730, part II: r = −0.637, part III: r = −0.728, part IV: r = −0.687). Although the SWCT scores in the current study (part I: r = 0.738, part II: r = 0.767, part III: ρ = 0.724, part IV: ρ = 0.673) were similar to those in a previous study (part I: r = −0.730, part II: r = −0.637, part III: r = −0.728, part IV: r = −0.687) [24], the TMT part B showed higher values than those in a previous study [24], indicating high validity. We assumed that this was because many patients in the previous study were excluded in the TMT part B (N = 96 to 56). In addition, Mok et al. [25] validated the total FAB score using the MMSE and Wisconsin Card Sorting Test (WCST). The results highlighted the validity of the total FAB score, with r values of 0.77 with the MMSE score, and −0.37 to 0.45 with the WCST score. The association between the total FAB score and the MMSE score in a previous study [25] was higher than that in the present study (ρ = 0.458). We assumed that this was because the total FAB score in the previous study was 8.9 ± 3.7 points [25], which is approximately 4 points lower than the value in the present study (total FAB score: 13.4 ± 2.8). Therefore, the present study indicates higher convergent validity.

In the current study, Cα was 0.92, and ICC (2.1) was 0.856 (95% CI: 0.761–0.915); these values indicate strong internal consistency and good reliability [21,22]. This result is consistent with the findings of previous studies [8,12,26]. The Japanese version of the FAB for dementia, which was developed by Kugo et al. [12], had a Cα of 0.715 and an ICC of 0.972. In addition, the Japanese version of the FAB for the frontal variant of frontotemporal dementia by Nakaaki et al. [12] had a Cα of 0.70 and an ICC of 0.89. The Cα was even lower in another study [27]. Therefore, the ICC (2.1) was similar to that in previous studies [12,26], but Cα was higher than that in previous studies [12,26,27]. The current study added additional modification in which FAB items 3 (Luria motor sequences), 4 (conflicting instructions), 5 (go-no-go test), and 6 (prehension behavior), which are related to movement, were to be performed on the non-paralyzed side. Previous studies did not specify which of these motor items were performed on which upper extremity [8,12,26]. We assumed that the Cα value was higher than that in previous studies because of the unification of the assessment. Therefore, the Japanese version of the FAB for patients with stroke indicated strong internal consistency and good reliability.

The study used the Bland–Altman analysis to check for proportional and fixed errors, and LOA were calculated because of the observed additive errors. The resulting LOA ranged from −1.7 to 2.9 points. The LOA indicate an acceptable range of measurement error, and the range identified in this study indicates a high probability of measurement error [13]. Any change in the value above this range indicated that the effect of rehabilitation or other interventions was greater than the measurement error. Manuli et al. [28] investigated whether virtual reality + robotic rehabilitation improved motor and executive functions, such as the FAB in patients with stroke. The results showed that the virtual reality + robotic rehabilitation group had significantly improved motor function and total FAB scores, with a difference of 5.4 points between the pre- and post-treatment scores. We assumed that the statistical results of the previous studies were not within the LOA. Hence, the results were beyond measurement error, and the study clearly demonstrated the effectiveness of rehabilitation.

This study has certain strengths and limitations. The strength of this study is that the FAB is applicable to patients with stroke and can be used to assess executive dysfunction. In addition, the LOA were calculated to determine intervention effects and errors. A limitation of this study is that an additive error was observed, indicating that the effect of recovery or learning effectiveness were contaminated because of subacute phase patients. Therefore, it is necessary to shorten the time period. Second, the other limitation is the small number of patients. Therefore, it is recommended that future studies be conducted with a larger number of patients and over a longer period of time. Thirdly, one previous study has assessed the reliability and validity of a shortened version of the FAB, known as the FAB-15, which excludes the subtest 6 based on a robust data-driven approach [29]. In future research, we aim to explore the validity of FAB-15 in stroke patients. Additionally, we plan to include a healthy control group in future studies to strengthen our experimental research plans. Finally, future studies should include a healthy group so that the FAB scores can be compared.

## 5. Conclusions

The Japanese version of the FAB showed strong internal consistency and good reliability for patients with stroke and is a useful assessment tool for capturing executive dysfunction in patients with stroke.

## Figures and Tables

**Table 1:** Characteristics of the participants.

Variables	Overall (N = 52)
Age (years)	72.6 ± 10.2 (48–89)
Sex (male/female)	32/20
BMI (kg/m^2^)	21.8 ± 4.2 (13.8–37.2)
Type of stroke (infarction/hemorrhagic)	31/21
Paretic side (right/left)	25/27
Time since stroke (days)	71.4 ± 63.8 (10–414)
SIAS for grip strength on the non-paralyzed side (points)	3 (0–3)
BRS upper limb	5 (1–6)
BRS finger	5 (1–6)
BRS lower limb	5 (1–6)
FIM motor (points)	64.5 ± 19.0 (17–91)
FIM cognitive (points)	28.4 ± 7.3 (3–35)
FIM total (points)	93.4 ± 22.5 (24–126)

Data are expressed as mean ± standard deviation (min-max) or median (min-max). BMI: Body Mass Index, SIAS: Stroke Impairment Assessment Set, BRS: Brunnstrom Recovery Stage, FIM: Functional Independence Measure.

**Table 2:** Results of the Frontal Assessment Battery and other assessments.

Assessments	Overall (N = 52)
FAB part 1 (points)	2.3 ± 0.6 (1–3)
FAB part 2 (points)	1.9 ± 0.7 (0–3)
FAB part 3 (points)	2.1 ± 0.8 (0–3)
FAB part 4 (points)	2.5 ± 0.7 (0–3)
FAB part 5 (points)	1.8 ± 0.9 (0–3)
FAB part 6 (points)	2.8 ± 0.4 (1–3)
FAB total (points)	13.4 ± 2.8 (7–18)
MMSE (points)	27.0 ± 4.8 (18–30)
TMT part A (s)	76.0 ± 40.4 (3–174)
TMT part B (s)	168.9 ± 117.2 (45–497)
TMT part B–A (s)	104.4 ± 93.1 (3–419)
SCWT part Ⅰ correct number	18.0 ± 8.7 (1–34)
SCWT part Ⅱ correct number	15.4 ± 8.4 (1–37)
SCWT part Ⅲ correct number	14.1 ± 7.6 (2–37)
SCWT part Ⅳ correct number	10.1 ± 7.5 (0–31)

Data are expressed as mean ± standard deviation (min–max) or median (min–max). FAB: Frontal Assessment Battery, MMSE: Mini-Mental State Examination, TMT: Trail Making Test, SCWT: Stroop Color Word Test.

**Table 3:** Results of convergent validity.

Assessments	Correlation Coefficients	*p* Value
MMSE	0.458	0.001
TMT part A	−0.389	0.005
TMT part B	−0.622	0.001
TMT part B–A	−0.614	0.001
SCWT part Ⅰ correct number	0.738	0.001
SCWT part Ⅱ correct number	0.767	0.001
SCWT part Ⅲ correct number	0.724	0.001
SCWT part Ⅳ correct number	0.673	0.001

MMSE: Mini-Mental State Examination, TMT: Trail Making Test, SCWT: Stroop Color Word Test.

## Data Availability

The datasets generated during and/or analyzed during the current study are available from the corresponding author on reasonable request.

## Supplementary Materials

The following Supporting Information can be downloaded at: https://www.mdpi.com/article/10.3390/neurolint16050081/s1, Documents S1: The Japanese version of FAB.
