# Supplementary material for: Validity and Reliability of the Japanese Version of the Frontal Assessment Battery in Patients with Stroke
Source: Neurol Int. 2024 Sep 25;16(5):1086–93. doi: 10.3390/neurolint16050081 (PMC11510384; doi:10.3390/neurolint16050081)
Supplement: Supplementary file 1 [file neurolint-16-00081-s001.zip › neurolint-3148717-supplementary.pdf]

Frontal Assessment Battery　脳卒中片麻痺患者 Version

日時＿＿＿＿＿＿＿＿

患者 ID:＿＿＿＿＿＿＿＿＿　患者名＿＿＿＿＿＿＿＿

1. 類似性： 概念化課題

「次の２つは，どのような点が似ていますか.」　　《反応》

① バナナ と オレンジ*　　【 果物 】（　　）

② テーブル と 椅子　　【 家具 】（　　）

③ チューリップ と バラ と ヒナギク　【 花 】（　　）

＊①のみヒント可：「どこも似ていない」など完全な間違いの場合や「どちらも皮がある」など部分的な間違いの場合は「バナナとオレンジはどちらも・・・」とヒントを出す．しかし点数は 0 点とする.②, ③はヒント無し.

【採点】 正答数 3: 3 点 正答数 2: 2 点 正答数 1: 1 点 正答数 0: 0 点　　（　　）点

2. 語の流暢性: 知的柔軟性課題

「"か"という字で始まる単語を出来る限りたくさん言って下さい．ただし人の名前と固有名詞は除きます.」

制限時間は 60 秒，最初の 5 秒間反応が無ければ，「たとえば紙」とヒントを出す.

10 秒黙っていたら「"か"で始まる単語なら　何でも良いですから」と刺激する.

＜反応＞

【採点】 １０語以上：３点 ６～９語：２点 ３～５語：１点 ２語以下：０点　（　　）点

3. 運動系列： 運動プログラミング課題

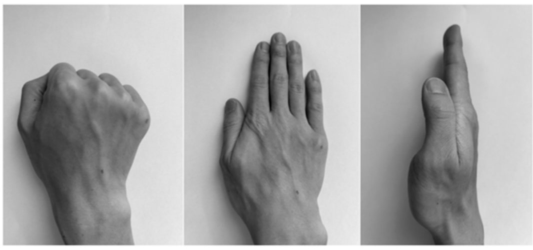

「私がすることをよく見ておいて下さい」

検査者は患者の前に座り，患者の非麻痺側と反対側でＬｕｒｉａの系列

「fist-edge-palm（拳 - 手刀 - 掌)」 を 3 回やって見せる.

そして「では，麻痺していない手で同じことをして下さい.最初は私と一緒に，次に一人でやってみて下さい」と言う．検査者は患者と一緒に 3 回繰り返し，その後「さあ，一人でやってみてください」と患者に言う.

＜備考＞

【採点】 患者 1 人で，正しい系列を 6 回連続してできる: 3 点

患者 1 人で，正しい系列を 3 回連続してできる: 2 点

患者 1 人では出来ないが，検査者と一緒なら正しい系列を 3 回連続してできる: 1 点

検査者と一緒であっても，正しい系列を 3 回連続することができない: 0 点

（　　）点

4. **葛藤指示: 干渉刺激に対する敏感さ・反応の選択課題**

「私が 1 回叩いたら, 2 回叩いて下さい」 患者が理解したことを確認して, 次の系列を試行する :1 - 1 - 1 「私が 2 回叩いたら，1 回叩いて下さい」 患者が理解したことを確認して，次の系列を試行する :2 - 2 - 2 そして次の系列を実施する ⇒ { 1 - 1 - 2 - 1 - 2 - 2 - 2 - 1 - 1 - 2 }

＜備考＞

*非麻痺側で実施する。

【採点】 間違い無し: 3 点

1，2 回の間違い: 2 点

3 回以上の間違い: 1 点

4 回以上連続して，検査者と同じ様に叩く: 0 点 （　　　）点

5. **GO/NO - GO: 抑制コントロール課題**

「私が 1 回叩いたら，1 回叩いて下さい」 患者が理解したことを確認して，次の系列を試行する :1 - 1 - 1

「私が 2 回叩いたら，叩かないで下さい」 患者が理解したことを確認して，次の系列を試行する :2 - 2 - 2

そして次の系列を実施する ⇒ { 1 - 1 - 2 - 1 - 2 - 2 - 2 - 1 - 1 - 2 }

＜備考＞

*非麻痺側で実施する。

【採点】 間違い無し: 3 点

1，2 回の間違い: 2 点

3 回以上の間違い: 1 点

4 回以上連続して，検査者と同じ様に叩く: 0 点 （　　　）点

6. **把握行動: 環境に対する被影響性**

検査者は患者の前に座り，患者の非麻痺側の手のひらを上に向けて，患者のひざの上に置く. 検査者はそれ以上何も言わずに自らの片手を患者の手の近くに持っていって非麻痺側の手のひらに触れる.そして患者が自発的に検査者の手を握るかどうかを見る. 患者が手を握ったら，次のように言ってもう一度繰り返す. 「今度は私の手を握らないでください」

【採点】 患者は検査者の手を握らない: 3 点

患者は戸惑って，何をすればいいのか尋ねてくる: 2 点

患者は戸惑うことなく，検査者の手を握る: 1 点

患者は「今度は，握らないで下さい」と言われた後でも検査者の手を握る: 0 点 （　　　）点

FAB 合計点 （　　　）/18 点
